# How and why patterns of sexual dimorphism in human faces vary across the world

**DOI:** 10.1038/s41598-021-85402-3

**Published:** 2021-03-16

**Authors:** Karel Kleisner, Petr Tureček, S. Craig Roberts, Jan Havlíček, Jaroslava Varella Valentova, Robert Mbe Akoko, Juan David Leongómez, Silviu Apostol, Marco A. C. Varella, S. Adil Saribay

**Affiliations:** 1 Department of Philosophy and History of Science, Faculty of Science, Charles University, Prague, Czech Republic; 2 Center for Theoretical Study, Charles University and Czech Academy of Sciences, Prague, Czech Republic; 3 Division of Psychology, University of Stirling, Stirling, FK9 4LA UK; 4 Department of Zoology, Faculty of Science, Charles University, Prague, Czech Republic; 5 Department of Experimental Psychology, Institute of Psychology, University of Sao Paulo, São Paulo, Brazil; 6 Department of Sociology and Anthropology, Faculty of Social and Management Science, University of Buea, Buea, Cameroon; 7 Human Behaviour Lab, Faculty of Psychology, Universidad El Bosque, Bogotá, Colombia; 8 Department of Anatomy, Animal Physiology and Biophysics, University of Bucharest, Bucharest, Romania; 9 Department of Psychology, Kadir Has University, Istanbul, Turkey

**Keywords:** Anthropology, Biological anthropology, Sexual selection

## Abstract

Sexual selection, including mate choice and intrasexual competition, is responsible for the evolution of some of the most elaborated and sexually dimorphic traits in animals. Although there is sexual dimorphism in the shape of human faces, it is not clear whether this is similarly due to mate choice, or whether mate choice affects only part of the facial shape difference between men and women. Here we explore these questions by investigating patterns of both facial shape and facial preference across a diverse set of human populations. We find evidence that human populations vary substantially and unexpectedly in both the magnitude and direction of facial sexually dimorphic traits. In particular, European and South American populations display larger levels of facial sexual dimorphism than African populations. Neither cross-cultural differences in facial shape variation, sex differences in body height, nor differing preferences for facial femininity and masculinity across countries, explain the observed patterns of facial dimorphism. Altogether, the association between sexual shape dimorphism and attractiveness is moderate for women and weak (or absent) for men. Analysis that distinguishes between allometric and non-allometric components reveals that non-allometric facial dimorphism is preferred in women’s faces but not in faces of men. This might be due to different regimes of ongoing sexual selection acting on men, such as stronger intersexual selection for body height and more intense intrasexual physical competition, compared with women.

## Introduction

Sexual dimorphism is among the most striking of phenomena across various species, our own species being no exception. Human sexual dimorphism is associated with many biological and psychological characteristics including sexual maturity, reproductive potential, mating success, general health, immune responses, sociosexuality, perceived age, and personality attributions^1–6^. Since Darwin, numerous proposals have been suggested to explain sexual dimorphism, the most popular of which is sexual selection.

The most extensively studied dimorphic trait in humans is facial dimorphism. While some sexually dimorphic facial traits are mediated by prenatal exposure to sex hormones^7^, dimorphism reaches full expression after puberty, due to the influence of androgens and estrogens^8,9^. High levels of testosterone are primarily responsible for development of masculine facial morphologies, while development of female secondary sexual traits is attributed to a high estrogen-to-testosterone ratio^10–14^.

Evidence from several vertebrate taxa indicates that testosterone may increase disease susceptibility due to negative effects on immune responsiveness^15^. Testosterone-dependent traits, such as male secondary sexual characters, are thus posited to signal health and physical fitness because only high-quality individuals can develop them^16–18^. We might therefore expect systematic preferences for males possessing testosterone dependent traits (but see^1,19–21^). In humans, there is some evidence that taller and physically stronger men with more masculine faces and voices are relatively healthy^22,23^.

For women, preference for higher levels of testosterone and concomitant masculinity might bring not only benefits of increased social dominance and immunological competence in a partner, but also potential costs in a resulting committed relationship, including risk of aggression and low paternal investment^24–26^. Studies in Western populations have reported varying effects, with some showing that women prefer facial masculinity in men^27–29^ and others the opposite, that is, a preference for relative facial femininity in male faces^30–32^. Male facial masculinity appears to be a trait preferred more strongly in some contexts or by some individuals, and reasons for such differences are not entirely clear. One explanation is that masculinity preferences might be influenced by temporal context, such that women seeking short-term partners prefer relatively masculine traits compared with those seeking long-term partners^33^. However, other evidence suggests the opposite, that masculinity preferences are stronger when seeking longer-term, co-parenting partners compared with short-term, sexual partners^34^. Self-assessed individual differences, such as one’s own condition, may also affect preferences^35^. On the other hand, the significance of context-dependent factors was recently challenged by a twin study showing that genetic differences explain vastly more variation in women’s preferences^36^.

In contrast, men’s preferences for female facial femininity show a much more systematic pattern. Although there is some research showing environment-related variation in facial femininity preferences^37^, more feminine female faces are perceived as more attractive unanimously across populations^12,30,38,39^. This may be because it could reveal reproductive potential (i.e. fecundity), as women with more feminine faces may have higher levels of estrogen^40^.

There are two contrasting views on cross-cultural variation in preferences of human sexual dimorphism. The first is that masculine traits signal characteristics that are adaptive and thus should be preferred in harsh environments (e.g., with resource scarcity, high disease prevalence, and pathogen load)^26^. For instance, Jamaican women exhibited a greater preference for masculine over feminine male faces compared to British women^41^. Furthermore, it has been indirectly suggested that masculine traits in women may be adaptive, for its possible advantages in heavy labor and social competition, in communities living in harsh environmental conditions^42–44^, including rural and traditional (pastoral and semi-nomadic, hunter-gatherer) societies. On the other hand, Scott et al.^19^ suggested that preference for sexually dimorphic traits is an evolutionarily novel feature which emerged in urban Western societies. According to this second view, masculine traits should be preferred only in developed societies with resource richness, and there should be no association between sexual dimorphism and attractiveness in traditional societies exposed to a relatively harsh environment.

The magnitude of sexual dimorphism may be affected by overall morphological variation of populations. The null preferences for masculine male faces in small-scale traditional societies were explained by encountering fewer different faces and by having fewer social interactions with potential mates over a lifespan^19^. A conjoint phenomenon might be the existence of lower variability of facial morphologies in less populated rural societies compared to higher facial variation in large-scale urban societies.

Facial morphology is affected by overall body size^45–48^, which is also sexually dimorphic and thus influences the perception of various social traits. Taller and heavier men are not only perceived as more masculine^49^ but taller men also possess more masculinized facial structure^20^. Height is also associated with male health. Tall (but not the tallest) men seem to have optimized immunity function^50^ . Maintaining body mass is costlier in regions with uncertain food availability. Greater mass may also be disadvantageous for hunting as it may make one more visible to prey; as indicated by the negative correlation between body size and food returns in African hunter-gatherers^51^. Himba nomads from northern Namibia showed preference for equal height instead of “taller male–shorter female” stereotype^43^. Likewise, women of the Tanzanian Hadza tribe showed no preference for large body size in potential mates^52^ and they were more likely to marry men shorter than themselves compared to British women^53^. Despite some exceptions, male tallness is preferred across human societies while there is no such simple preference for female height^54–57^. In this study, we generated a large dataset (N = 1317) of facial portraits to explore how facial dimorphism varies across eight human populations (Brazil, Cameroon, Colombia, Czech Republic, Namibia, Romania, Turkey, and United Kingdom). Although there is anatomical and anthropological evidence that dimorphism in the craniofacial complex varies across various human populations^20,58–61^, our study more directly addresses patterns of face shape and its perception, investigating how the observed patterns of sexual dimorphism are related to the overall face shape variation, local differences in stature between sexes, variation due to size (allometry), and mate preferences for faces. Specifically, we address the following research questions:

(i) What are the differences in face sexual shape dimorphism (SShD) across various human populations?
(ii) How are these differences influenced by allometric and non-allometric variation in SShD? The critical question is how sexual dimorphism interacts with body size in different human populations and what the preferences for size-dependent variation in facial shape are. If facial dimorphism is systematically associated with body size variation across populations, one would expect less facial dimorphism in populations with less dimorphism in body height.
(iii) How does the morphological variation in face and body height affect the differences in SShD across populations? Higher morphological variation may be a necessary precursor to a higher degree of sexual dimorphism. We therefore expect that populations with lower levels of facial shape variation will be less sexually dimorphic. Further we inspected the role of body height differences between sexes to overall variation of SShD across populations.
(iv) What are the preferences for facial sexual dimorphism across populations? Due to women’s preference for taller men, allometric sexually dimorphic cues in men’s faces should be preferred. At the same time, we expect that allometric masculinity is linked with facial appearance, reflecting less negative personality traits than non-allometric masculinity. Hence, we expect allometric masculinity to be preferred. The evolutionary reason may be the optimization of the human male phenotype, combining prosocial facial traits and greater body size. In female faces, we expect the opposite pattern: non-allometric femininity should be associated with facial attractiveness because no systematic association between female facial attractiveness and body height is expected.

## Materials and methods

### Acquisition of facial photographs

We used 1317 standardized frontal photographs, some of which were used in previous studies^62–67^. The number of individuals in each population is shown in supplementary table S1 and demographic characteristics of the studied populations are provided in supplementary table S4. For purposes of allometric decomposition, the dataset was restricted to 1114 individuals for whom we had reliable information about their body height (summarized in supplementary table S3). The facial images were taken by standardized protocol within each population, which allowed a subsequent measure of sexual dimorphism. All participants were asked to adopt a neutral, non-smiling expression, and to remove facial cosmetics, jewelry, or other decorations, if possible. We instructed participants to look directly into the camera to avoid vertical and horizontal head tilts. The photographs were subsequently post-produced to adjust the eyes horizontally at the same height.

To demonstrate that the variation in focal length does not pose a problem for deriving our conclusions, we regress facial shape on logarithm of focal length and considered only the residuals of this regression as a material for alternative analysis with equivalent regression models and summarizations. The results of this alternative analysis are very similar to the results of the main analysis that does not account for focal length (see https://osf.io/sn56z/).

### Attractiveness ratings

Rating sessions took place in each of the investigated populations, and raters judged only opposite-sex faces from their own population. Ratings were collected using images presented on a computer screen. Raters from all populations (except Colombia) were asked to judge the attractiveness of 50 faces of the opposite sex on a 7-point verbally anchored scale (from “1—not at all attractive” to “7—very attractive”). In Colombia, attractiveness was scored on a 0.0 to 10.0 scale (with one decimal place), anchored verbally from “0.0—not at all attractive” to “10.0—very attractive”. Facial images were presented in a randomized order and time spent rating was not restricted. The ratings for each face were averaged and scaled (mean = 0, SD = 1) by population before analysis. All raters were also asked to report their age and nationality; for details about the sample sizes and descriptive statistics about raters from particular populations, see supplementary table S2.

### Geometric morphometrics

For each of 1317 faces, we defined 72 landmark positions, from which 36 were a posteriori indicated as semi-landmarks. Landmarks are homologous points that usually correspond to well-defined anatomical and morphological facial structures and can thus be unambiguously identified across all faces in the sample. Semi-landmarks (or sliding landmarks) were used to quantify two-dimensional homologous outlines and curvatures of facial morphology that could not be characterized as traditional landmarks^68^. See supplementary figure S1 for the positions of landmarks and semilandmarks.

The measurement error was estimated on the subsample of 400 faces. The landmarks were placed manually on each facial image by two persons trained by the first author. All configurations were also visually inspected by the first author before analysis. We have employed procD.lm function from the geomorph package to execute the analysis of variance between individual faces and within each individual face landmarked my multiple digitizers (included in a model as an effect of the interaction between the digitizer and the individual) or by a single digitizer twice. Proportion of the variation accountable to the landmarked face is reported as measurement repeatability. The overall repeatability was calculated from the subsample of 400 faces: within digitizer repeatability was estimated on the subsample of 200 faces that were landmark by the same digitizer twice and the between digitizer repeatability estimated on the subsample of 200 faces that were independently landmarked by two digitizers. A repeatability of digitizing precision between two replicates was 0.951 (measurement error: 0.048). The within digitizer repeatability estimated on the subsample of 200 faces that were landmarked by the same digitizer twice was 0.963, the between digitizer repeatability estimated on the subsample of 200 faces that were landmarked by two digitizers was 0.929. The distribution of facial data was checked for possible digitizing errors due to landmark application and outliers by visual inspection of PCA plots and by using plotOutliers function in the Geomorph package in R^69^. PCA was employed by gm.prcomp function in the Geomorph package. Any outliers which were due to a digitizing error were detected prior to analysis and corrected.

All shape coordinates were superimposed by generalized Procrustes analysis (GPA) using the gpagen function in the Geomorph package in R^69,70^. Semi-landmark positions were optimized based on minimal bending energy criterion. After semi-landmarks were slid, aligned coordinates were symmetrized; that is, left and right sides were reflected along the midline and mirrored configurations were then averaged using the symmetrize function in the Morpho package^71^.

We measured morphological disparity, estimated as Procrustes variance, to compare morphological variation among groups of faces defined by sex and population. To test for differences in morphological disparity between groups, the morphol.disparity function in the Geomorph package was used, with significance testing based on 9,999 permutations.

Shape variation associated with sexual shape dimorphism of all examined groups were visualized using thin-plate spline (TPS) deformation grids^72,73^. All thin-plate spline extrapolations, and combined plots, were performed with use of the plotRefToTarget function in the Geomorph R package^69^.

### Calculating the degree of sexual shape dimorphism of the face

Sexual Shape Dimorphism (SShD) was computed by projection of the individual facial configurations in facial morphospace onto the vector between male and female means. This vector method, i.e. using group averages to define an axis of morphological differences between men and women, has been applied in numerous previous studies on human sexual dimorphism^20,66,74–76^. The position of an individual’s face (A) along the axis connecting male (MM) and female mean (FM) shape can be expressed as a dot product of a vector from the origin to the coordinates of A and a vector from FM to MM, i.e.

$$ sexscore\left( {\vec{A}} \right) = \vec{A} \cdot \overrightarrow {{\left( {MM - FM} \right)}} $$

Higher negative scores indicate more female-like morphology, whereas higher positive scores indicate a more male-like facial shape. To visualize differences in both magnitude and direction of SShD vectors in multidimensional morphospace, we conducted a trajectory analysis using the RRPP R-package^70^.This overall measure of SShD can be decomposed to allometric and non-allometric components, i.e. to variation in SShD that is due to body size (allometric) and variation that is independent of size (non-allometric). Body height was used as a measure of an individual’s size. The allometric variation in SShD was calculated by regressing the original facial coordinates on height and projecting the estimated values from this regression on the vector of sex differences. The non-allometric component of SShD was acquired by regressing the original shape coordinates on height and then projecting the residualized facial coordinates on the sex difference vector calculated on these residuals.

The female scores of overall, allometric, and non-allometric SShD were inverted (multiplied by − 1). Higher values represents more masculine faces in the case of men and more feminine faces in women.

The angle $\alpha$ between the vector of overall SShD $\overrightarrow {{v_{1} }} = \left( {MM - FM} \right)$ and its allometric component ($\overrightarrow {{v_{2} }} =$ vector of coefficients of multiple regression of facial shape on body height) and between $\overrightarrow {{v_{1} }}$ and its non-allometric component $\overrightarrow {{v_{3} }} =$ ($MM_{res} - FM_{res}$) were calculated from the ratio of a dot product of given vectors and a product of their norms following formulas

$$  cos\left( \alpha  \right) = \frac{{\overrightarrow {{v_{1} }}  \cdot \overrightarrow {{v_{2} }} }}{{\left\| {\overrightarrow {{v_{1} }} } \right\|\left\| {\overrightarrow {{v_{2} }} } \right\|}},\quad cos\left( \nu  \right) = \frac{{\overrightarrow {{v_{1} }}  \cdot \overrightarrow {{v_{3} }} }}{{\left\| {\overrightarrow {{v_{1} }} } \right\|\left\| {\overrightarrow {{v_{3} }} } \right\|}}. $$

### Statistical procedures

Linear mixed effect models of the influence of overall, allometric, and non-allometric SShD on average rated attractiveness were conducted using the lmer function from the lmerTest package^77^. Females were used as the reference category, and we report the slope of the regression of attractiveness on SShD. The difference between male and female intercepts and male and female slopes were included in the regression model, as fixed effects, to test the difference between males and females. Fully specified random slopes and intercepts by population were included in the model as random effects.

Pearson product moment correlation coefficients were used for all correlational analyses. These were calculated using a cor.test and cor functions from base R.

The regression relationships on populational levels (e.g., difference in body height vs the distance between male and female mean shapes, or the difference or morphological disparity versus SShD between sexes) were evaluated with Bayesian regression with vague weakly informative priors (normal distributions with mean = 0 and SD = 1 on a linear model with standardized variables). The models were fitted with quap function from the rethinking package on standardized variables. The regression estimates and the compatibility corridors were sampled from the posterior distribution using the link function from the rethinking package^78^.

To weight calculated descriptive statistics against their null distributions, two permutation tests were conducted: (1) Randomization test, where populations are assigned at random to facial shapes, while the gender assignment of each face and the number of men and women in each sample remain unchanged; and (2) Random split sample test, where each sample was divided into two random subsamples of equal size, and then distribution of average differences between subsamples from the same populations was compared with the distribution of average differences between subsamples from different populations. Ten thousand randomized samples were generated within each permutation test.

### Ethics statement

All the experiment protocol for involving humans was in accordance to guidelines of national/international/institutional or Declaration of Helsinki. This study does not include information or images that could lead to identification of a study participant.

### Informed consent

Informed consent was obtained from all participants. All procedures mentioned and followed were approved by the Institutional Review Board of the Faculty of Science of the Charles University (protocol ref. number 06/2017).

## Results

### Variation in sexual shape dimorphism across populations

**Figure 1:**
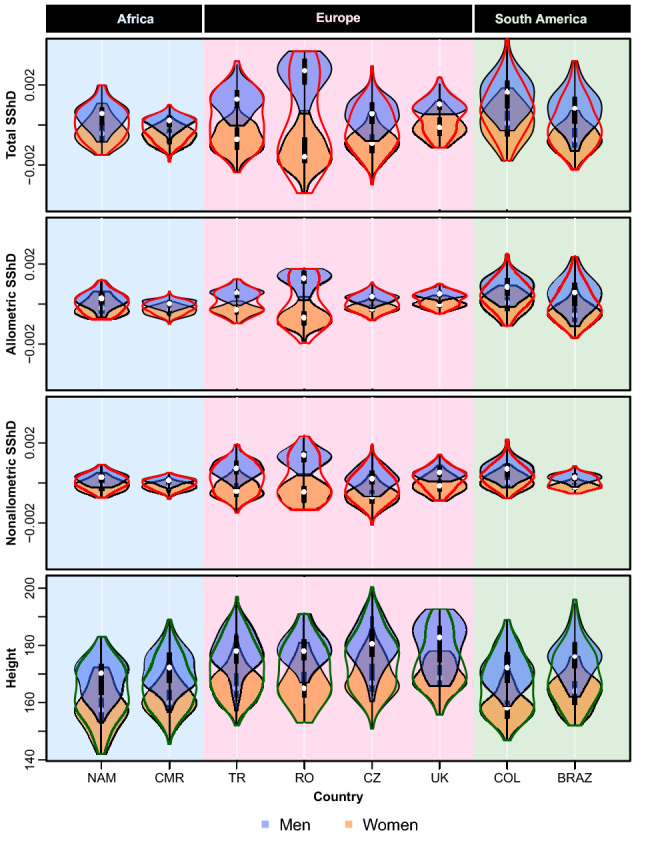
Violin plots showing range and variation in sexual shape dimorphism (SShD; overall, allometric, and non-allometric) and body height across eight populations (NAM-Namibia, CMR-Cameroon, TR-Turkey, RO-Romania, CZ-Czechia, UK-United Kingdom, COL-Colombia, BRAZ-Brazil). White points indicate medians, black rectangles represent interquartile ranges.

Populations differed in the degree of sexual shape dimorphism measurable from the face (SShD). Romanians showed the greatest range of sexually dimorphic facial traits. Cameroonians showed the lowest SShD, followed by the Namibian faces (see Fig. 1, and supplementary figure S2 for the full dataset). African populations revealed a lower level of facial shape dimorphism (mean distance between distribution medians = 0.00089) as compared to Europeans (mean = 0.00224) and South Americans (mean = 0.00158). The probability (yielded by permutation test with randomized populational labels) that the difference between African mean distance between sex-specific medians and the equivalent characteristics of other populational samples (0.00202) emerged by chance alone was p < 0.001 (expected difference was 0.001, SD = 0.0001); see supplementary figure S3.

**Figure 2:**
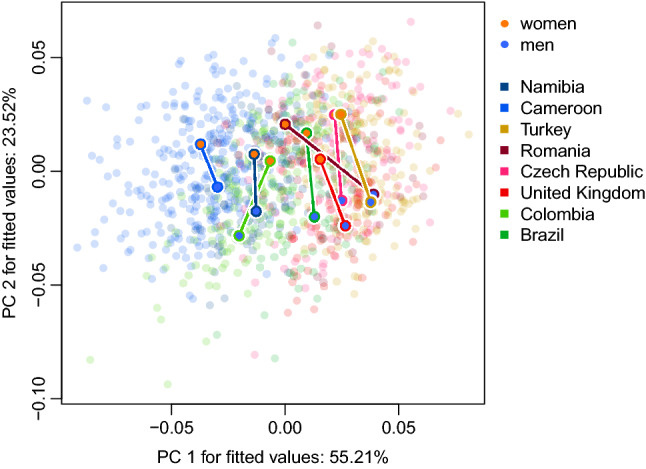
The results of trajectory analysis on the first two principal components. PC1 captures mostly the variation between national samples with European samples on the right and African samples on the left, while PC2 allows faces to align along sexual dimorphism axis. Both differences in direction and magnitude contribute to differences in face shape sexual dimorphism in our samples.

The trajectory analysis (see Fig. 2) revealed that PC1 mostly captures the variation between population samples with European samples on the right and African samples on the left, while PC2 allows faces to align along the sexual dimorphism axis. Both differences in direction and magnitude contribute to differences in face shape sexual dimorphism in our samples (see tables S6 and S7 for pairwise differences between studied samples).

When we filter out the effect of body weight on the facial shape (See https://osf.io/ydkat/), the main results stay almost unchanged. Here, we report the analysis without accounting for body weight because that allowed us to include the data from United Kingdom, where the weight of female targets was not known. The raw sexscores and sexscores after conditioning on body weights were nearly identical (Pearson’s r = 0.92 for overall sexscores, r = 0.86 for allometric, and r = 0.93 for the non-allometric component of SShD, see Supplementary material Figure S11).

### Differences in allometric and non-allometric components of SShD across populations

**Figure 3:**
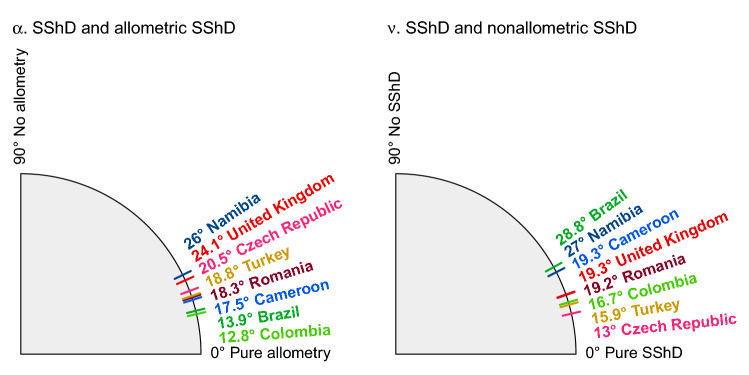
Angles between total SShD vector separating male and female means and its allometric (α) and non-allometric components (ν).

The decomposition of overall SShD into allometric and non-allometric components (see Figs. 1, 3), revealed that contribution of these to overall facial dimorphism also differs across populations.

To examine this more precisely, we measured the angle between the total SShD vector and its allometric component (α), and the angle between the total SShD vector and its non-allometric component (ν). A lower angle indicates a higher dependence of the SShD on a given component. The results are visualized in Fig. 3. The total SShD is essentially projected onto the allometric and non-allometric vectors. Therefore, the ratio of the angles is inversely proportional to the ratio of total standard deviations in given components (r = 0.98). This visualization is complementary to violin plots in Fig. 1 and shows the order of the SShD level across the populations. Accordingly, body size is strongly related to facial dimorphism in the Brazilian and Colombian samples, while non-allometric variation in SShD contributes to sexual dimorphism especially in the Czech, Turkish, and Colombian samples. Both allometric and non-allometric SShD contribute similarly to sex differences in Cameroon, Namibia, Romania, and Colombia.

The correlation between *α* and *ν* was very low (r = 0.07), which indicates that both components contribute to the total SShD independently. The angles between allometric and non-allometric vectors (effectively *α* + *ν*, see supplementary table S5) were lower than 90° in all samples, which means that both components of human SShD point in a similar direction in the multidimensional space constituted by facial coordinates. The split sample test showed that the expected difference between $\alpha$ in two subsamples from the same population was 8.30, *SD* = 2.3, while the average difference between populations was 8.55, *SD* = 1.40 (*p* = 0.94). The expected difference between $\beta$ in two subsamples from the same population was 8.31, *SD* = 2.43, while the average difference between populations was 9.10, *SD* = 1.43 (*p* = 0.62). The variation between angles may be due to chance and needs to be interpreted with caution (see supplementary figure S4).

**Figure 4:**
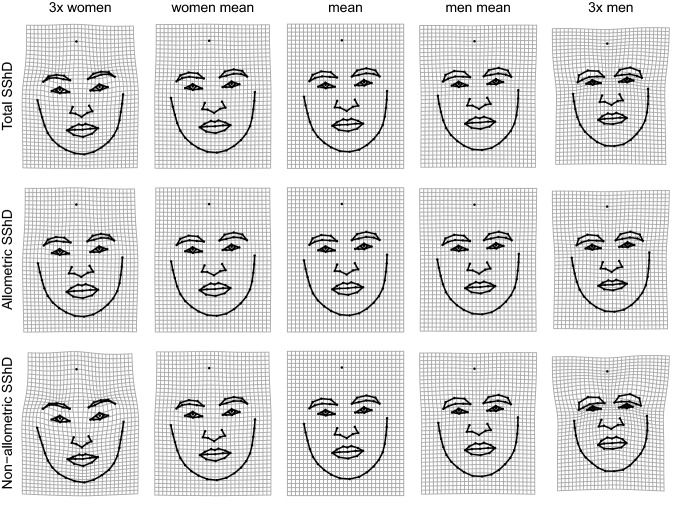
Thin-plate spline visualizations of facial shape variation associated with differences between sexes and its decomposition to allometric and non-allometric components. Thin-plate splines are shown within observed range and 3 × extrapolated compared to an average configuration in the middle.

**Figure 5:**
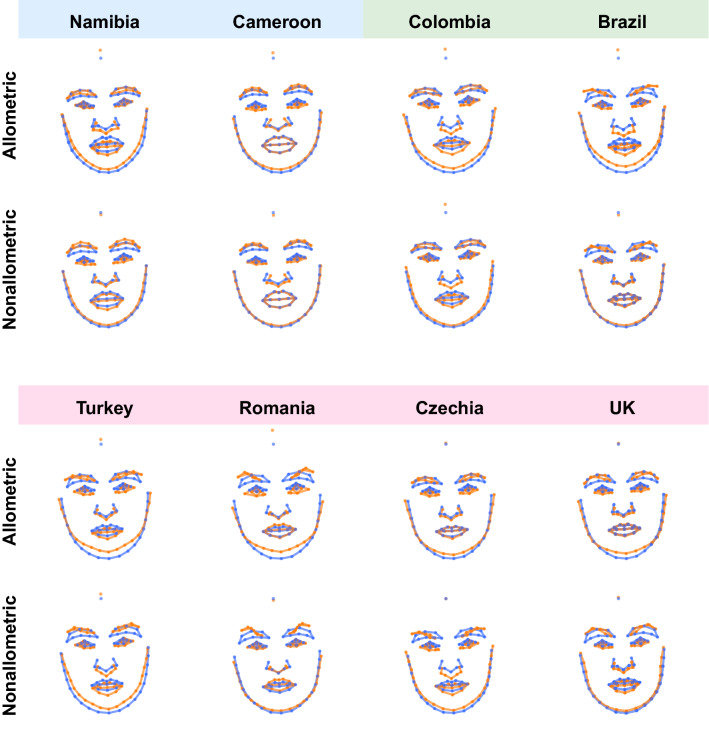
The depiction of allometric and non-allometric shape differences between male (blue) and female (orange) averages shown separately for each population. The shape differences were 5 × magnified for better interpretability.

The differences between male and female facial shapes based on faces from all eight populations are shown in Fig. 4 (see supplementary figures S5-S7 for sex differences shown separately for each population). The upper panel shows overall shape sexual dimorphism, while the two lower panels depict allometric and non-allometric components. The traits associated with female facial morphology are characterized by a rather gracile structure with a smaller nose, mouth with fuller lips, bigger eyes ornamented by thinner brows. In contrast, male facial structure is generally more robust, with greater lower face area, a bigger lower jaw with the rounded chin, smaller eyes closer to pronounced brows, and a bigger nose with broader radix. When decomposed into allometric and non-allometric variation, the facial shape differences between men and women appeared rather weakly linked with allometric variation. In contrast, non-allometric variation, i.e., shape changes independent of size, revealed strong shape differences (grid dilations/constriction), especially in the area of the eyes and lower face, between men and women. Figure 5 depicts the shape difference between male and female facial configurations for allometric and non-allometric components separately for all populations.

### The effects of morphological variation in face and body height on SShD

#### Body height differences predict morphological distance between sex averages but not the overall variation in facial shape dimorphism

**Figure 6:**
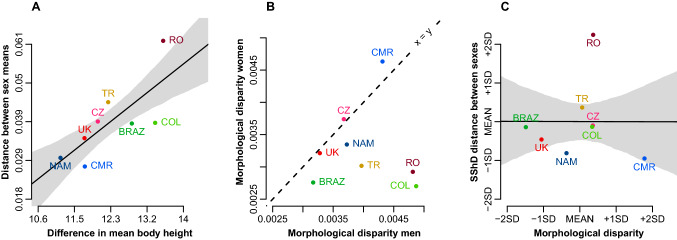
Relationship between difference in sex-specific average statures (x-axis) and average morphological distance between male and female face (y-axis). The difference in body height predicts the distance between the mean facial shape of men and women. All European countries have higher facial dimorphism than expected (**A**). Faces of men are morphologically more variable than faces of women (**B**); total SShD is independent of morphological disparity (**C**). Shaded bounds indicate 89% compatibility corridors of the regression line.

The relationship between sex differences in mean stature and distance between mean sex-specific facial configurations across all examined populations is shown in Fig. 6A. The sex difference in body height predicts the distance between male and female facial averages. However, this does not mean that variation in body size itself explains the variation across populations in facial shape dimorphism (e.g., range of SShD). Europeans showed greater morphological differences between sex-specific facial means than expected from differences in stature, while South Americans showed unexpectedly tight proximity of sex-specific means.

#### Did morphological variation in human face drive the evolution of sex differences?

Morphological disparity of men (MD_men_ = 0.0042) was significantly higher (*p* < 0.001) than morphological disparity of women (MD_women_ = 0.0037); see also Fig. 6B. This was true even when morphological variation in the examined populations was taken into account (MD_men_ = 0.0032, MD_women_ = 0.0030, *p* = 0.026). We also measured the morphological disparity of each population; see Table 1. All pairwise differences higher than 0.0008 were statistically significant (0.006 for the morphological disparity accounting for sex). Male and female morphological disparity across populations was not correlated (*r* =—0.01). Total morphological disparity was not associated with SShD (Fig. 6C).

**Table 1:** Morphological disparity (MD) by population.

	NAM	CMR	TR	RO	CZ	UK	COL	BRAZ
MD total	0.0037	0.0045	0.0038	0.004	0.004	0.0034	0.0039	0.0032
MD without sex variation	0.0035	0.0044	0.0034	0.0035	0.0036	0.0032	0.0038	0.0029
MD men	0.0037	0.0043	0.004	0.0048	0.0037	0.0033	0.0049	0.0032
MD women	0.0033	0.0046	0.003	0.0029	0.0037	0.0032	0.0027	0.0028

### Preferences for facial sexual dimorphism across populations

**Figure 7:**
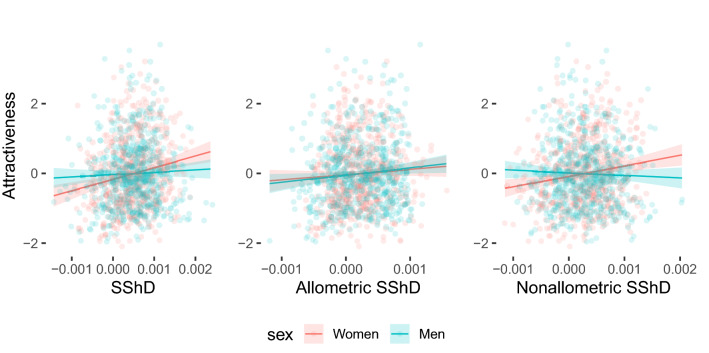
The relationship between overall, allometric, and non-allometric sexual shape dimorphism (SShD) and rated facial attractiveness.

The relationship between attractiveness ratings and facial sexual dimorphism was examined with linear mixed effect models. Female faces with higher overall, allometric, and non-allometric SShD scores were rated as more attractive. However, this relationship did not hold for male faces in overall and non-allometric component of SShD. Only more masculine male faces in allometric SShD (i.e., faces of taller men) were rated as more attractive than less masculine faces. The regression slope for male and female faces did not differ in the allometric component. See Table 2 for the complete results of the fixed effect estimations in all three models. The model results are visualized in Fig. 7 (for the specific situation in each population, see supplementary figures S8-S10).

**Table 2:** Linear mixed effect model results.

	Estimate	Std. Error	df	t value	Pr( >|t|)
**Overall SShD and attractiveness**					
Intercept (women)	0.001	0.042	918.5	0.03	0.976
SShD slope (women)	0.19	0.045	15.7	4.18	0.001
Change in intercept of man	0	0.059	955.8	0.01	0.995
Change in slope of man	− 0.155	0.063	7.5	− 2.45	0.042
**Allometric SShD and attractiveness**					
Intercept (women)	0.079	0.045	145.9	1.75	0.082
SShD slope (women)	0.153	0.067	8.8	2.26	0.05
Change in intercept of man	− 0.024	0.092	6.2	− 0.27	0.799
Change in slope of man	− 0.003	0.115	6.9	− 0.03	0.979
**Non-allometric SShD and attractiveness**					
Intercept (women)	0.002	0.042	17.5	0.04	0.971
SShD slope (women)	0.151	0.043	2	3.47	0.075
Change in intercept of man	− 0.001	0.06	81.6	− 0.02	0.984
Change in slope of man	− 0.188	0.06	4.4	− 3.11	0.031

## Discussion

Human populations vary in both magnitude and direction of sexually dimorphic facial traits (see Figs. 1, 2, and tables S6-S7). People of European origin and South Americans showed a higher level of sexual dimorphism in facial shape compared to people of African origin. Eye region—the shape and relative size of eyes and brows (supraorbital ridge)—differed the most between sexes.

The possible explanation of lower sexual dimorphism in Africa may be either relaxed selection pressure favoring morphological femininity of female faces or masculinity of male faces, alternatively due to positive selection for more feminine faces in males, or the masculinization of faces in females. Our study cannot decide between these alternatives and future research should investigate which of these hypotheses is more plausible.

Face shape may not be the only characteristic that provides information about the level of sexual dimorphism in African populations. Lighter skin color has been shown to be an important cue of female attractiveness, potentially revealing youthfulness and residual fertility^65,79,80^. Preference for lighter skin tones is also reported in traditional Asian societies^81,82^. It is thus possible that the lower level of morphological sex difference reported here may be compensated by color cues to femininity in populations that exhibit highly variable skin color^79,83,84^. Moreover, preference for feminine-looking women has been found to be positively correlated with level of national health^37^. The relaxed preferences for femininity have been also previously reported in Jamaica and Bangladesh^41,85^. However, without knowledge of local range and distribution of facial sexual dimorphism in both shape and color, it is not clear whether the local men tend to prefer morphologically less feminine women or whether their preferences simply exploit non-shape components of female sexually dimorphic traits.

Furthermore, the extent to which sex differences in facial shape can be related to body size, and the contribution of shape variation due to size, seem to vary across populations (Fig. 1). Note however, that these differences in the relative contribution of allometric and non-allometric may be idiosyncratic to this dataset and need to be interpreted with caution.

One relevant question is whether these differences in facial dimorphism could be derived from sex differences in body height. Populations with greater sex differences in body size would tend to also have higher levels of facial dimorphism due to allometry. Although we have reported an association between sex differences in body height and distance between sex-specific facial averages (see Fig. 6A), this does not explain the variation either of total SShD or its allometric component. The differences in height between men and women across populations were negligible compared to facial sex differences. An interesting finding is that all European countries revealed larger differences between male and female average faces than in all the other populations. Again, this was true only for differences between sex averages but not for the overall range of sexual dimorphism. These differences in sex averages may indicate the result of (disruptive) sexual selection acting on European populations. But why do South American populations have lower distance between sex averages and still a very broad range of SShD? This might be due to genetic introgressions, ecological adaptation, or a combination of both. Admixture of genes from less dimorphic African populations, in combination with European and genuine Amerindians, increased genetic variation of recent South American populations^86,87^, which may be a reason for their wide range of SShD. Our South American samples consisted pervasively of individuals of recent mixed ethnical origin. Importantly, genetic drift rather than (or together with) selection might have played a role in shaping the population variation of the facial morphology, as was shown for example in the neurocranial and facial diversification of early *Homo* and modern humans^88,89^. Most probably, the cranial diversification results from a set of different processes, such as gene flow, environmental effects, and selection^90^.

Previous anthropometric studies have suggested a link between facial traits and climate^91–93^. Facial width tends to increase in wetter regions with a uniform climate, while nose height tends to decrease with air moisture^93^. This correlation between facial morphology and climatic conditions might indicate that facial sexual dimorphism in African tropical and subtropical populations is reduced due to morphological adaptation to climate. The wider noses of peoples inhabiting hot-wet environments may be an adaptation to expiratory heat elimination and brain cooling^94–97^. The higher magnitude of facial shape transformation along the vector of sexual dimorphism, as demonstrated by European faces, might be incompatible with climatic adaptations, such as the architecture of broader and shallower noses of African tropical populations.

Altogether, differing preferences for facial sexual dimorphism across countries do not explain the reported patterns of SShD. The association between sexual shape dimorphism and attractiveness is moderate for women and weak (or none) for men. One may argue that these associations might in fact be stronger if we further specify the attractiveness ratings such as sexual or long-term partner attractiveness. While we agree that such approach is useful when working within a specific society, the same cannot be said for cross-cultural research with highly variable types of relationships^98,99^. Based on two competing perspectives, the more masculine men should be either preferred in harsher environments (vs favorable environments) or in large-scale urban societies (vs small-scale rural societies). However, the morphological masculinity in faces of men was not preferred in any of the investigated populations. Despite the differences in the degree of facial sexual dimorphism in mostly rural (African) and pervasively urban societies (European and South American), there were no differences in facial masculinity preferences.

Moreover, the lack of preference for masculinity in rural societies might be due to reduced morphological variability in faces which results in the reduced opportunity to discern a possible association between behavior and sexually dimorphic morphology. This was not the case in our sample, as we showed that the morphological variability of African faces was not statistically different compared to the faces of European origin. Based on our results, we are not able to support either hypothesis based on environmental harshness or the level of societal development. However, the reader should take caution that a more direct test of the role of environment on facial preferences would ideally involve more fine-grained data (e.g., district-level environmental indicators), a worthy direction for future research.

Human male mating and reproductive adaptations are more associated with dominance and status, while female adaptations are strongly linked with signals of reproductive quality and health to attract mates^100^. This might explain why there is a consistent preference for facial femininity in women while no such preference exists for facial masculinity in men. Nevertheless, the perception of dimorphism is also influenced by variation due to size (allometry). On the one hand, the taller men have an advantage in terms of social status and dominance; on the other hand, facial correlates of body size may be responsible for negative personality impressions, such as perceived aggressiveness and dominance^24,30^. Hence, sexual selection should compromise between the benefits of tallness and negative personality inferences from facial morphology. In particular, tall men with prosocial, non-aggressive looking faces might be preferred by women. Our finding that male facial features associated with allometric dimorphism appeared to be less linked with negative personality traits compared to non-allometric dimorphism, supports this prediction. However, we did not bring direct evidence on the association between facial attractiveness and male allometric SShD. In women, where body height is not a decisive criterion in mate choice, feminine facial features that are independent of allometry are preferred.

Our work has several limitations. Despite the large total number of faces, some populations may be undersampled once we omitted individuals for whom data on body height was lacking. However, this does not affect the reported patterns of SShD. Future research would benefit from including an even larger number of populations—controlling for phylogenetic, ecological, and sociocultural confounding effects—in order to disentangle the causes of variation in facial SShD across various human populations. Further, the allometric component varies depending on whether centroid size, height, or weight is used as the measure of size^101^. We used body height as a measure of size because height plays an important role for mate choice and facial allometry reflecting the body height is likely to influence facial perception. Finally, it is worth noting that sexual dimorphism measured from facial morphology may not capture all visual aspects of sex differences to which humans are optically sensitive. Future studies using the direct measurement approach should thus combine the shape analysis with information involving sex differences in soft tissues, such as skin texture, complexion color, and so on.

## Conclusion

Recent studies have challenged the influential idea that sexual dimorphism of the human face reveals the biological quality of the face-bearer or affects their offspring's survival^20,102,103^. In this study, we presented new evidence about patterns and distribution of sexually dimorphic facial traits from several human populations across four continents. Building on previous evidence, we showed that the magnitude and direction of facial dimorphism varies substantially among human populations. Overall, morphological variability of the face does not seem to explain the variation in the pattern of facial dimorphism across populations, and sex differences in stature provided only partial insight. Finally, we investigated whether human facial shape dimorphism is correlated with attractiveness judgments, as a proxy of individual’s mate value. Only morphological femininity in women’s faces affected men’s attractiveness judgments, while masculinity in men’s faces was not consistently associated with female attractiveness judgments.

## Supplementary Information

Supplementary Information.

## Data Availability

The dataset and R code are available at https://doi.org/10.17605/OSF.IO/9DYBW.
