# Supplementary material for: How and why patterns of sexual dimorphism in human faces vary across the world
Source: Sci Rep. 2021 Mar 16;11:5978. doi: 10.1038/s41598-021-85402-3 (PMC7966798; doi:10.1038/s41598-021-85402-3)
Supplement: Supplementary file 1 — Supplementary Information. [file 41598_2021_85402_MOESM1_ESM.pdf]

Supplementary materials

How and why patterns of sexual dimorphism in human faces vary across the world

Karel Kleisner[1]*, Petr Tureček[1,2], S. Craig Roberts[3], Jan Havlíček[4], Jaroslava Varella Valentova[5], Robert Mbe Akoko[6], Juan David Leongómez[7], Silviu Apostol[8], Marco A. C. Varella[5], S. Adil Saribay[9]

[1] Department of Philosophy and History of Science, Faculty of Science, Charles University, Prague, Czech Republic
[2] Center for Theoretical Study, Charles University and Czech Academy of Sciences, Prague, Czech Republic
[3] Division of Psychology, University of Stirling, Stirling FK9 4LA, UK
[4] Department of Zoology, Faculty of Science, Charles University, Prague, Czech Republic
[5] Department of Experimental Psychology, Institute of Psychology, University of Sao Paulo, Sao Paulo, Brazil
[6] Department of Sociology and Anthropology, Faculty of Social and Management Science, University of Buea, Buea, Cameroon
[7] Human Behaviour Lab, Faculty of Psychology, El Bosque University, Bogotá, Colombia
[8] University of Bucharest, Department of Anatomy, Animal Physiology and Biophysics
[9] Department of Psychology, Kadir Has University, Istanbul, Turkey

*corresponding author: karel.kleisner@natur.cuni.cz

Table S1. Sample sizes of facial photograph subsets

	NAM	CMR	TR	RO	CZ	UK	COL	BRAZ
N total	99 (90)	301	259 (184)	100 (48)	220 (154)	100 (99)	138	100
N men	49 (48)	150	115 (91)	50 (17)	110 (76)	50 (49)	72	49
N women	50 (42)	151	144 (93)	50 (31)	110 (78)	50 (50)	66	51

The sizes of restricted samples with reliable measures of body height are in brackets. NAM-Namibia, CMR-Cameroon, TR-Turkey, RO-Romania, CZ-Czechia, UK-United Kingdom, COL-Colombia, BRAZ-Brazil

Table S2. Sample sizes and descriptive characteristic of raters

	NAM		CMR		TR		RO	
raters' sex	M	F	M	F	M	F	M	F
N of raters	30	30	126	131	66	66	33	63
Mean Age	22,13	21,47	22,9	22,7	22,1	22,1	27,36	23,19
SD Age	4,69	4,2	4,2	3,2	3,7	3,7	7,29	4,32
Age Range	18-31	18-33	17-45	17-33	14-54	14-54	18-51	17-40
ICC	0,86	0,81	0,94	0,96	0,95	0, 96	0,95	0,97

	CZ		UK		COL		BRAZ	
raters' sex	M	F	M	F	M	F	M	F
N of raters	33	89	10	11	95	95	22	27
Mean Age	28,2	27,56	22,1	23,2	22,0	21,9	23,6	23,8
SD Age	4,21	4,23	2,02	2,18	3,92	4,81	3,65	3,87
Age Range	18-40	19-47	19-25	21-27	17-59	17-59	18-30	18-30
ICC	0,97	0,99	0,98	0,85	0,95	0,92	0,93	0,92

N of raters - number of raters per single image, ICC - intraclass correlation coefficient, NAM-Namibia, CMR-Cameroon, TR-Turkey, RO-Romania, CZ-Czechia, UK-United Kingdom, COL-Colombia, BRAZ-Brazil

Table S3. Mean Age, height and weight of male and female faces ± 1SD

	NAM	CMR	TR	RO	CZ	UK	COL	BRAZ
Full sample								
Age men	23±3.6	22.6±3.9	21.8±2.2	23.3±3.9	22.5±3.5	20.9±3.4	20.4±2.4	23.3±3.6
Age women	23.3±3.9	23±5.9	21.6±1.6	22.6±4.5	22±3.4	21.4±3.4	20.7±2.8	25.7±11.5
Restricted sample								
Age men	23±3.6	22.6±3.9	21.5±1.9	23.9±4.6	23±3.6	20.7±2.9	20.4±2.4	23.3±3.6
Age women	23.2±3.9	23±5.9	21.2±1.5	21.4±2	22.6±3.8	21.4±3.4	20.7±2.8	25.7±11.5
Height men	168.8±6.6	172.1±6.5	178.3±7.2	179.2±5.9	180.4±7.7	180.3±7.9	172.2±6.6	176.1±7.1
Height women	157.7±7.4	160.4±5.5	166±5.8	165.7±7	168.4±7	168.6±5.3	158.9±5.9	163.3±5.7
Weight men	56.2±10.9	69.5±8.7	75.3±13.4	75.3±10.5	73.3±9.7	75.8±9.3	68.1±9.9	70.9±12.9
Weight women	55.9±16.4	64.8±13.7	57.2±8.8	53.9±8	62.5±8.4	NA	57.1±8.9	57.4±8.4

The age summaries were calculated for the full (N=1317) and the restricted (N=1114) sample with reliable measures of body height and weight. Summaries of height and weight were evaluated for the restricted sample only. NAM-Namibia, CMR-Cameroon, TR-Turkey, RO-Romania, CZ-Czechia, UK-United Kingdom, COL-Colombia, BRAZ-Brazil

Table S4. Demographic characteristics for studied populations

Country	NAM	CMR	TR	RO	CZ	UK	COL	BRAZ
Urbanization (%)	46.90	54.58	73.61	53.89	73.48	82.63	79.76	85.77
Population density (people per km^2)	2.81	49.29	102.04	86.57	137.25	272.23	42.83	24.46
Population size (thousands)	2315	23298	78529	19925	10601	65860	47521	204472
GDP per capita (US$)	5033	1327	10949	8978	17716	44966	6176	8814

Data on population size and density were extracted from World Population Prospects (UN, 2019), Urbanization data from World Urbanization Prospects (UN, 2018), and data on GDP from World Bank national accounts data (World Bank and OECD, 2019). Data are reported for the year 2015, the last year for which all precise information was available. Data are visualized below.

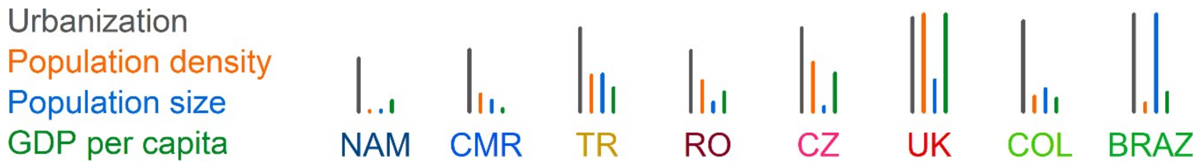

Visual representation of data in table S4 scaled by maximum value in each variable

United Nations, Department of Economic and Social Affairs, Population Division (2019). World Population Prospects – 2019 revision (available at https://population.un.org/wpp/, accessed on 23.1.2020)

United Nations, Department of Economic and Social Affairs, Population Division (2018). World Urbanization Prospects 2018 (available at https://population.un.org/wup/, accessed on 23.1.2020)

World Bank national accounts data, and OECD National Accounts data files (2019). GDP per capita (current US$) 2019 (available at https://data.worldbank.org/indicator/NY.GDP.PCAP.CD, accessed on 23.1.2020)

Table S5. The angles between the total maleness-femaleness vector and allometric and nonallometric maleness-femaleness vectors

	NAM	CMR	TR	RO	CZ	UK	COL	BRAZ
α	26.03	17.52	18.77	18.3	20.51	24.15	12.82	13.89
ν	27.02	19.28	15.88	19.16	12.96	19.25	16.7	28.83
α+ν	53.05	36.8	34.65	37.46	33.47	43.4	29.52	42.72
τ	52.89	35.85	32.54	34.49	33.02	42.91	28.73	42.19

α = the angle between the total maleness-femaleness and its allometric component, ν = the angle between the total maleness-femaleness and its nonallometric component. τ = the measured total angle between the allometric and non-allometric maleness-femaleness (this should be approximately equal to the sum of α and ν), CMR-Cameroon, TR-Turkey, RO-Romania, CZ-Czechia, UK-United Kingdom, COL-Colombia, BRAZ-Brazil

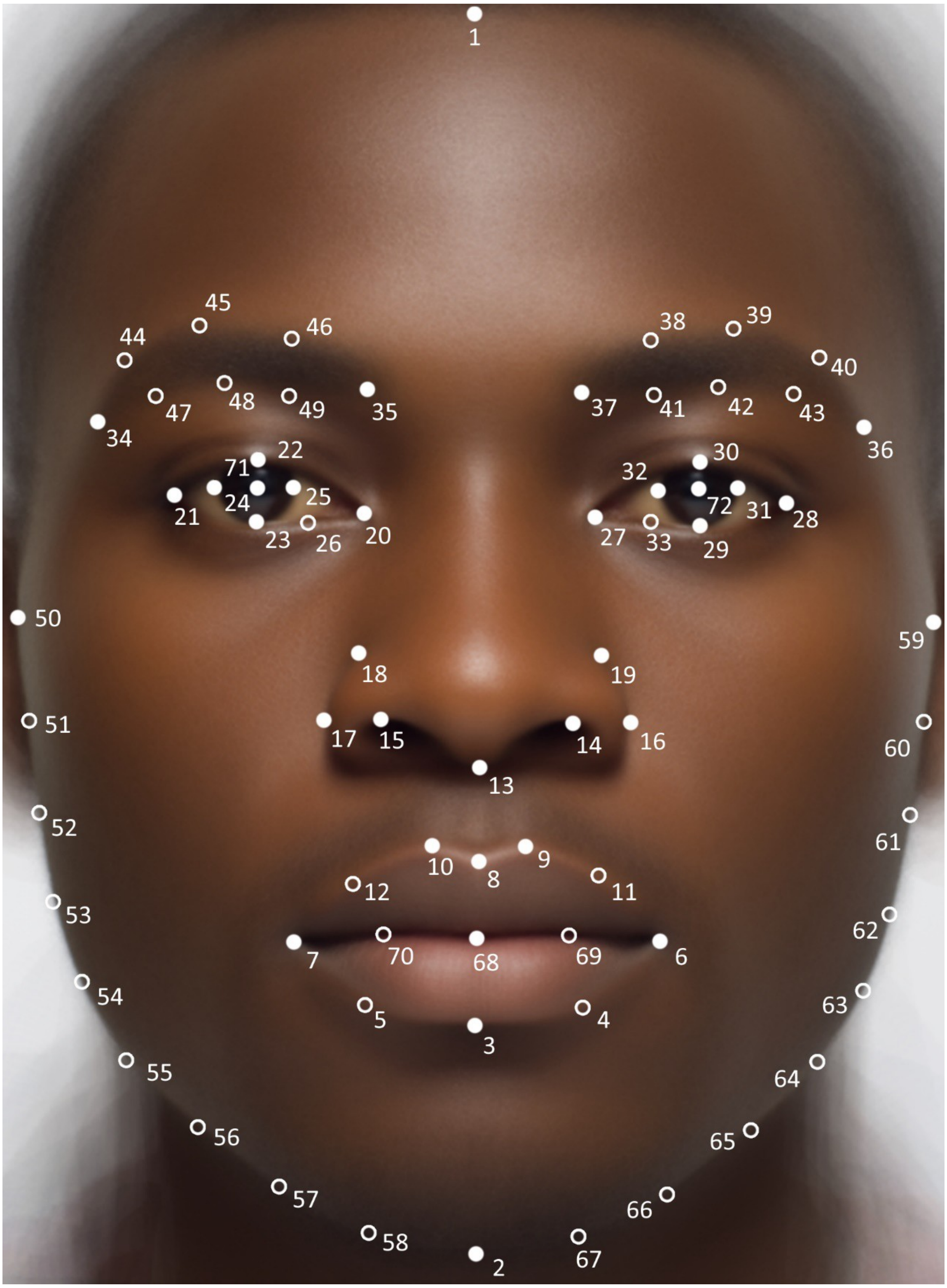

1	TRICHION	midpoint of the hairline, that is, on the hairline through the midline of the forehead
2	MENTON	the lowest point of the lower border of the mandible (along the jaw line)
3	LABIALE INFERIUS	the midline point of the lower vermilion line (border of the lower lip)

4,5		the midpoints between LABIALE INFERIUS (3) and CHEILON (9,10)
6,7	CHEILON	the outer corner of the mouth where the outer edges of the upper and lower lip meet
8	LABIALE SUPERIUS	the upper midpoint of the upper vermilion line, a point ofmaximum local curvature between the christae philtri
9,10	CHRISTA PHILTRI	the point on the crest of the philtrum, the vertical groove in the median portion of the upper lip on the vermilion border
11,12		the midpoints between LABIALE SUPERIUS (8) and CHRISTA PHILTRI (9,10)
13	SUBNASALE	midpoint of the angle at the columella base where the lower border of the nasal septum and the surface of the upper lip meet
14,15	COLUMELLA APEX	highest point of the columella crest at the apex of the nostril
16,17	ALARE	the most lateral point on the ala contour
18,19	ALAE ORIGIN	the most posterolateral point of the curvature of the base of the nasal alae
20,27	ENDOCANTHION	the inner corner of the eye fissure where eyelids meet
21,28	EXOCANTHION	the outer corner of the eye fissure where eyelids meet
22,30	PALPEBRALE SUPERIUS	the highest visible point of the iris
23,29	PALPEBRALE INFERIUS	the lowest visible point of the iris
24,31	Iris Outer Border	the rightmost point of the right iris (leftmost of the left iris)
25,32	Iris Inner Border	the leftmost point of the right iris (rightmost of the left iris)
26,33		the midpoint between ENDOCANTHION (20,27) and PALPEBRALE INFERIUS (23,29)
34,36	SUPERCILIARE LATERALE	the most lateral point of the eyebrow
35,37	SUPERCILIARE MEDIALE	the most medial point of the eyebrow
38-40,44-46	the eyebrow upper curve	three semilandmarks with regular spacing between SUPERCILIARE LATERALE (34,36) and SUPERCILIARE MEDIALE (35,37)
41-43,47-49	the eyebrow lower curve	three semilandmarks with regular spacing between SUPERCILIARE LATERALE (34,36) and SUPERCILIARE MEDIALE (35,37)
50,59	ZYGION	the most lateral point of the zygomatic arch
51-58,60-67	the lower jaw	eight semilandmarks with regular spacing between MENTON (2) and ZYGION (50,59)
68	STOMION	center of the lip crack, lying on the midline between LABIALE SUPERIUS (8) and LABIALE INFERIUS (3)
69,70		the midpoints between STOMION (68) and CHEILON (6,7)
71,72	Pupil	center of the pupil

Figure S1: Positions of 72 landmarks and semilandmarks on human face. The figure depicts an artificial composite face. The composite face was created using TpsSuper 2.05 software.

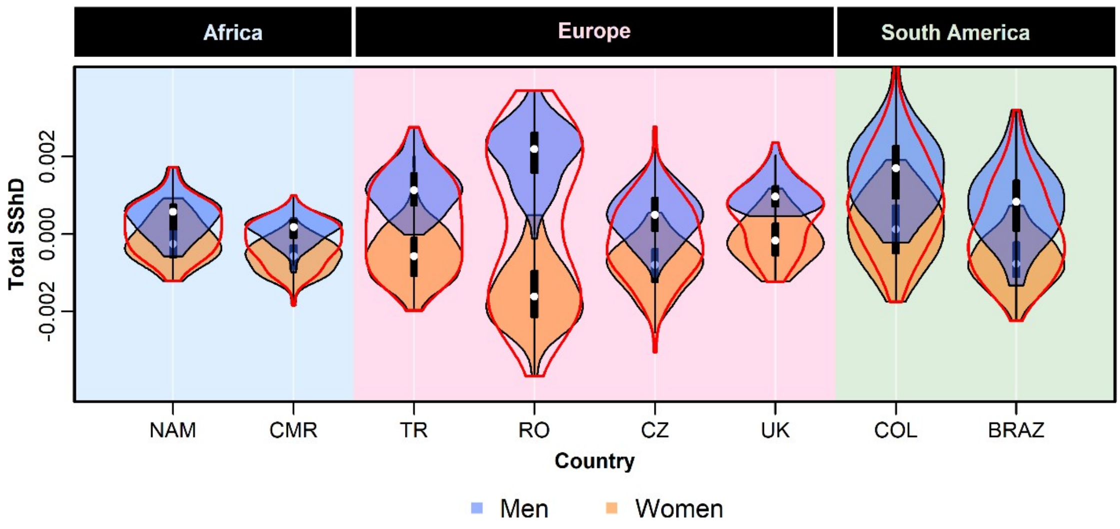

Figure S2: Violin plot showing range and variation in overall SShD evaluated in the full sample (1317 individuals). The violin plots for allometric and nonallometric components can be constructed only using the data where the information about body height is available. This panel (very similar to first panel in figure 1 in the main article) is presented here to demonstrate that the differences between the full sample and the restricted sample are minor, if any (NAM-Namibia, CMR-Cameroon, TR-Turkey, RO-Romania, CZ-Czechia, UK-United Kingdom, COL-Colombia, BRAZ-Brazil).

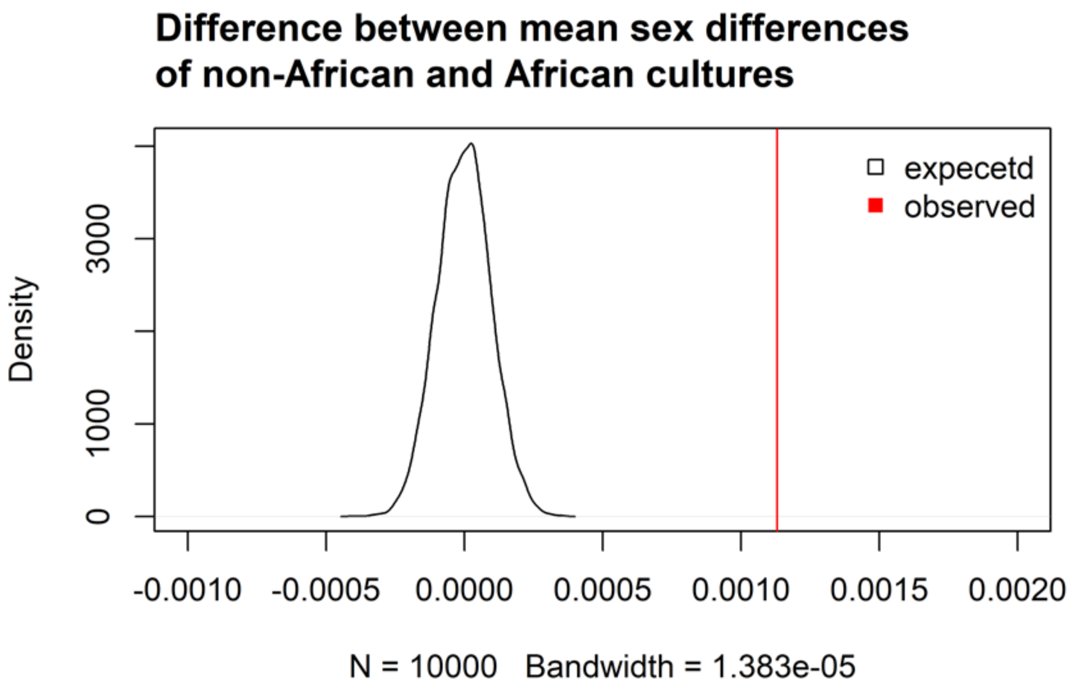

Figure S3. Randomization permutation test results. Comparison of the expected distributions of differences between African SShD mean European and South American SShD. Facial shape dimorphism in Africa is conclusively lower as compared to SShD in other cultures that would be expected by chance.

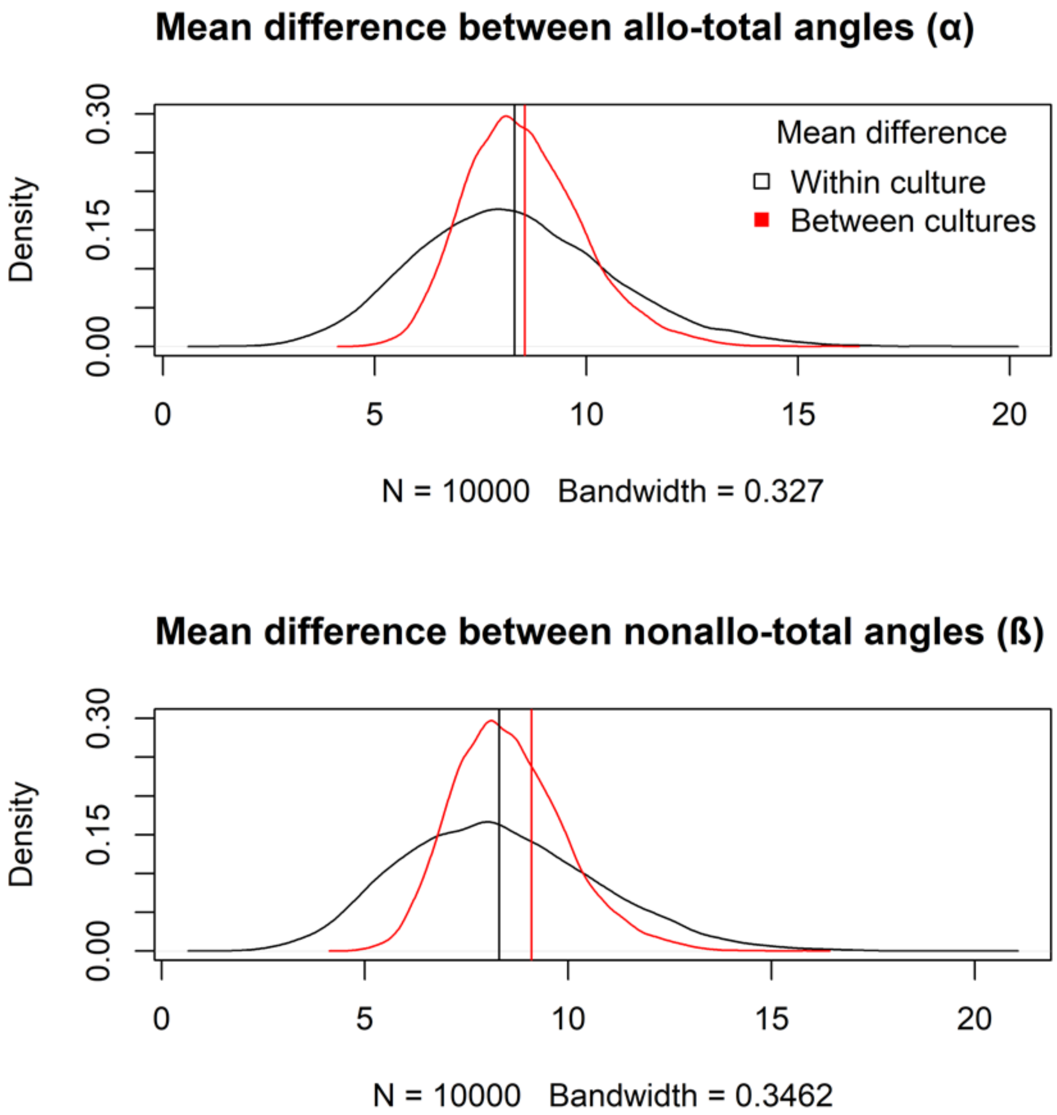

Figure S4. Split sample permutation test results. Comparison of the expected distributions of differences between angles from the same national sample and differences between angles from subsamples drawn from different national samples revealed that the variation in angles (α SD=4.56, β SD=5.43) might have arisen by chance.

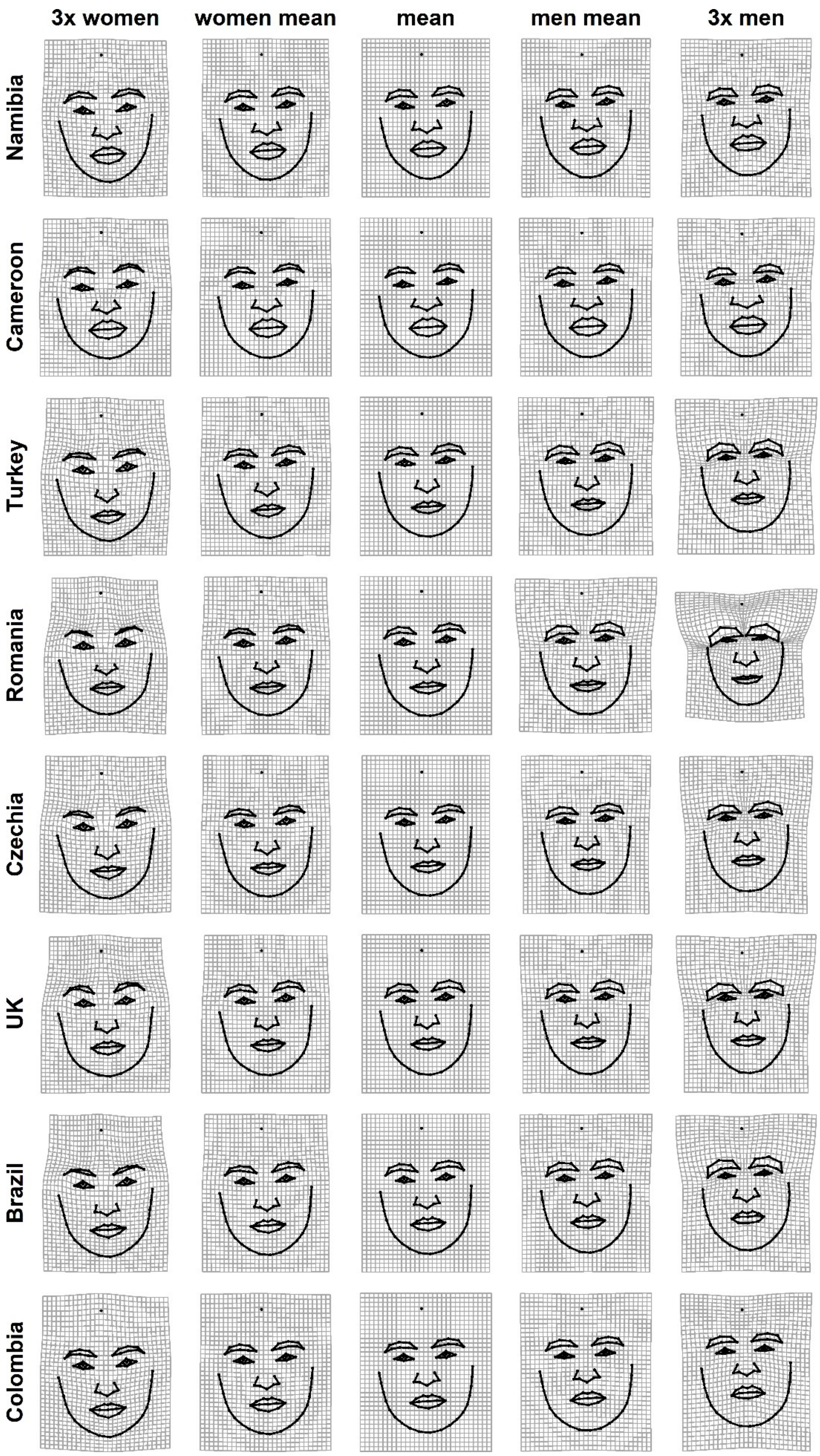

Figure S5. Thin-plate spline visualizations of facial shape variation associated with differences between sexes for each population shown within observed range and 3x extrapolated compared to an average configuration in the middle

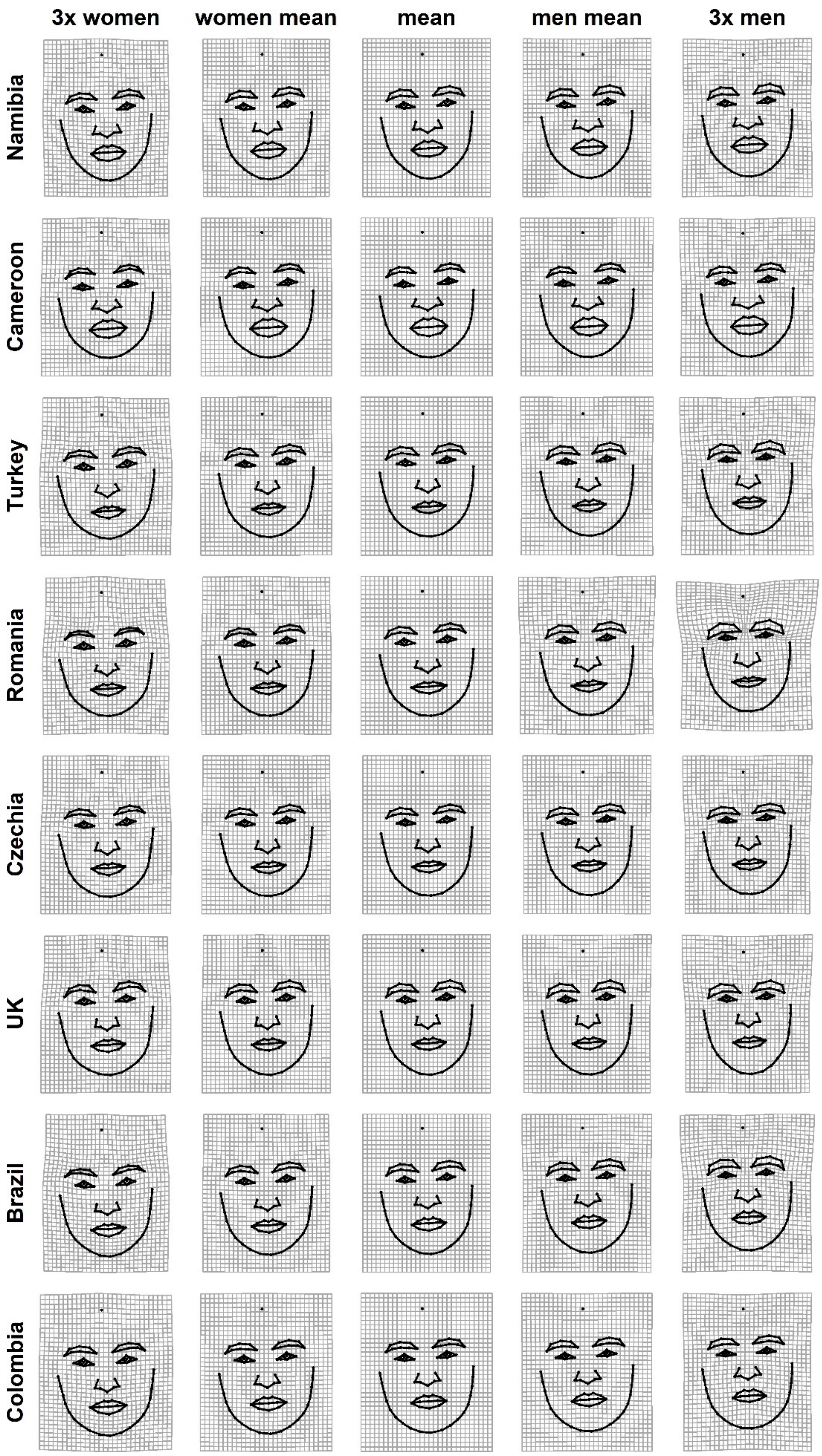

Figure S6. Thin-plate spline visualizations of facial shape variation associated with allometric differences between sexes for each population shown within observed range and 3x extrapolated compared to an average configuration in the middle

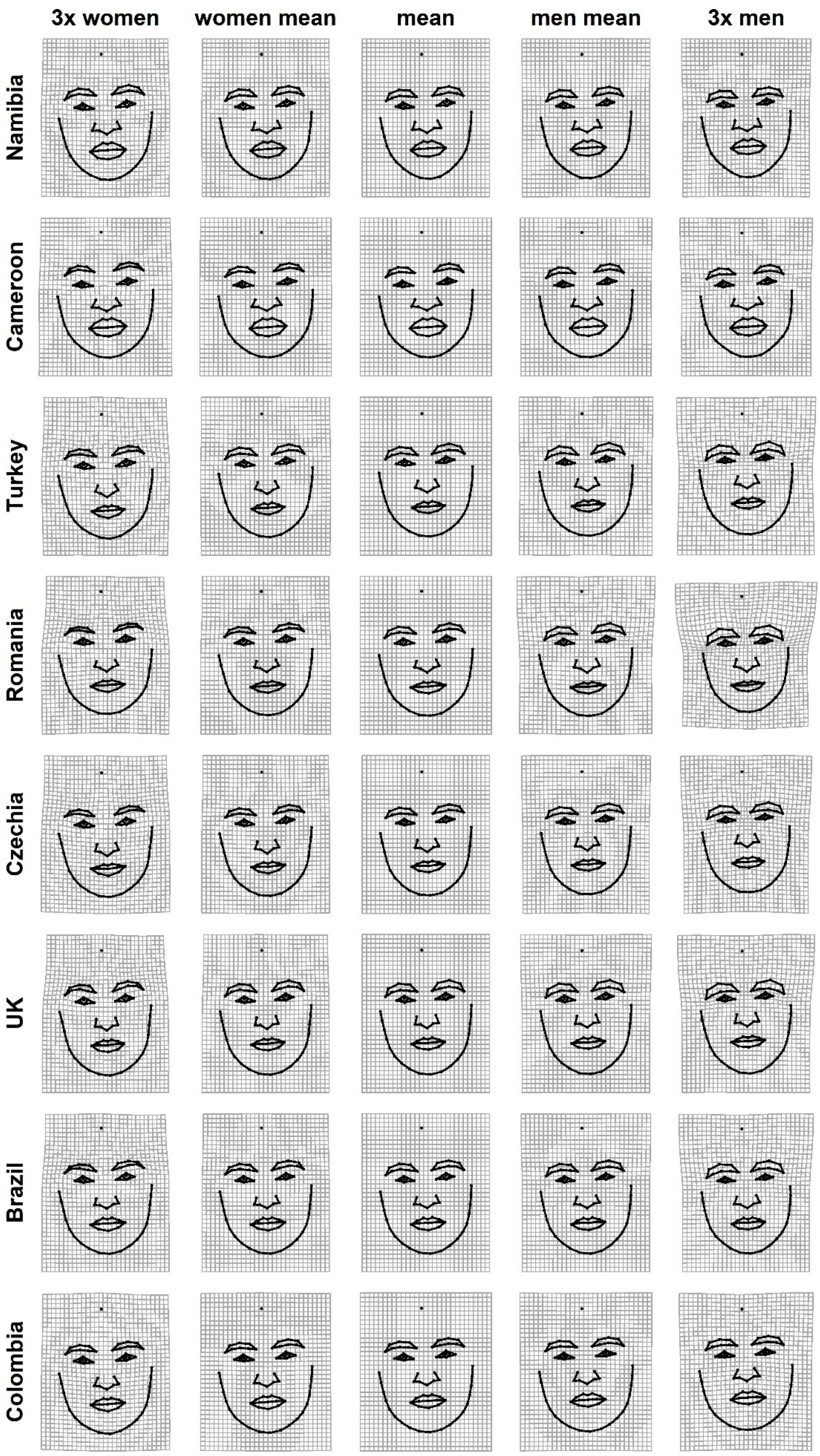

Figure S7. Thin-plate spline visualizations of facial shape variation associated with nonallometric differences between sexes for each population shown within observed range and 3x extrapolated compared to an average configuration in the middle

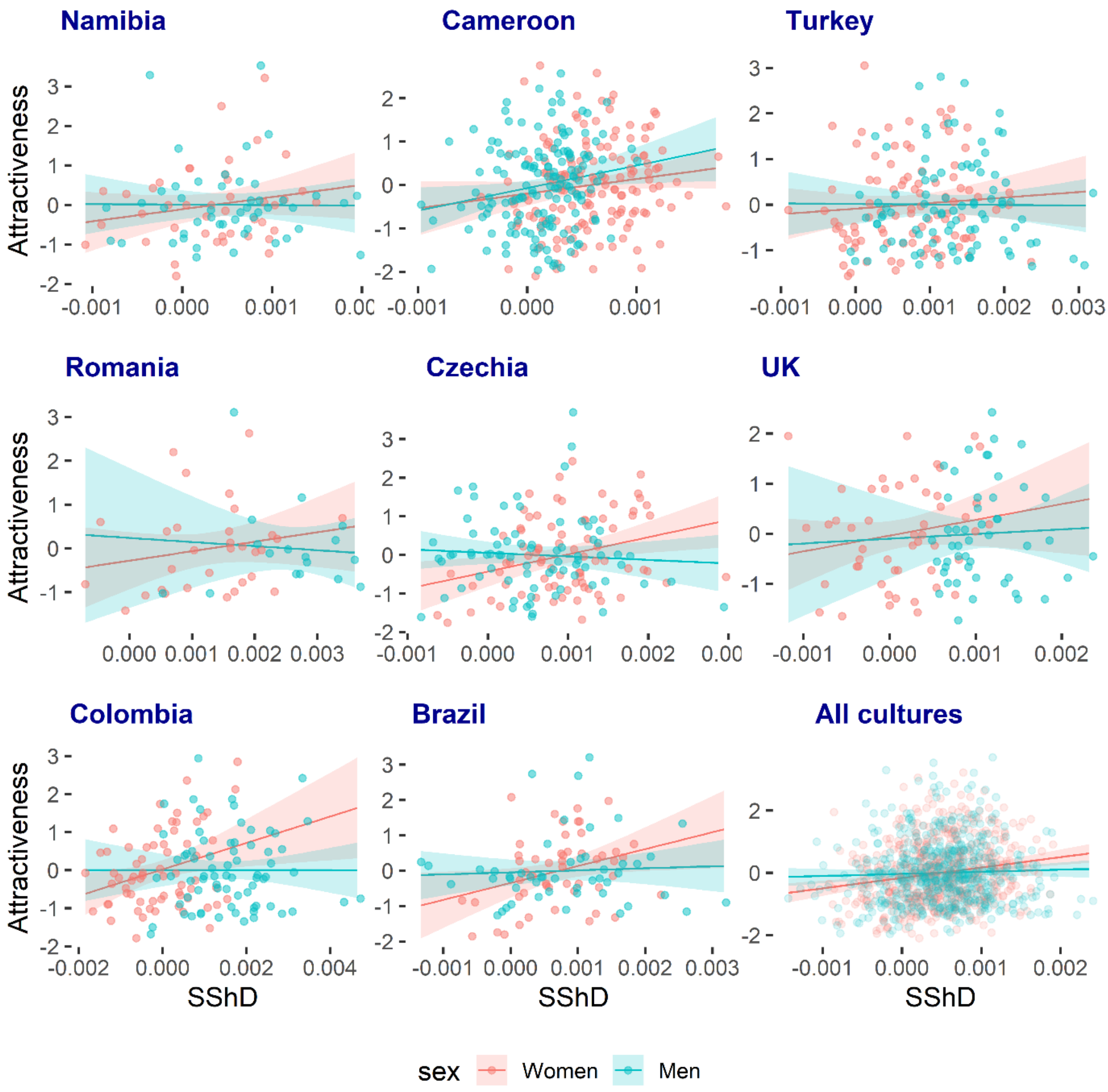

Figure S8. The relationship between total SShD and rated facial attractiveness in each population

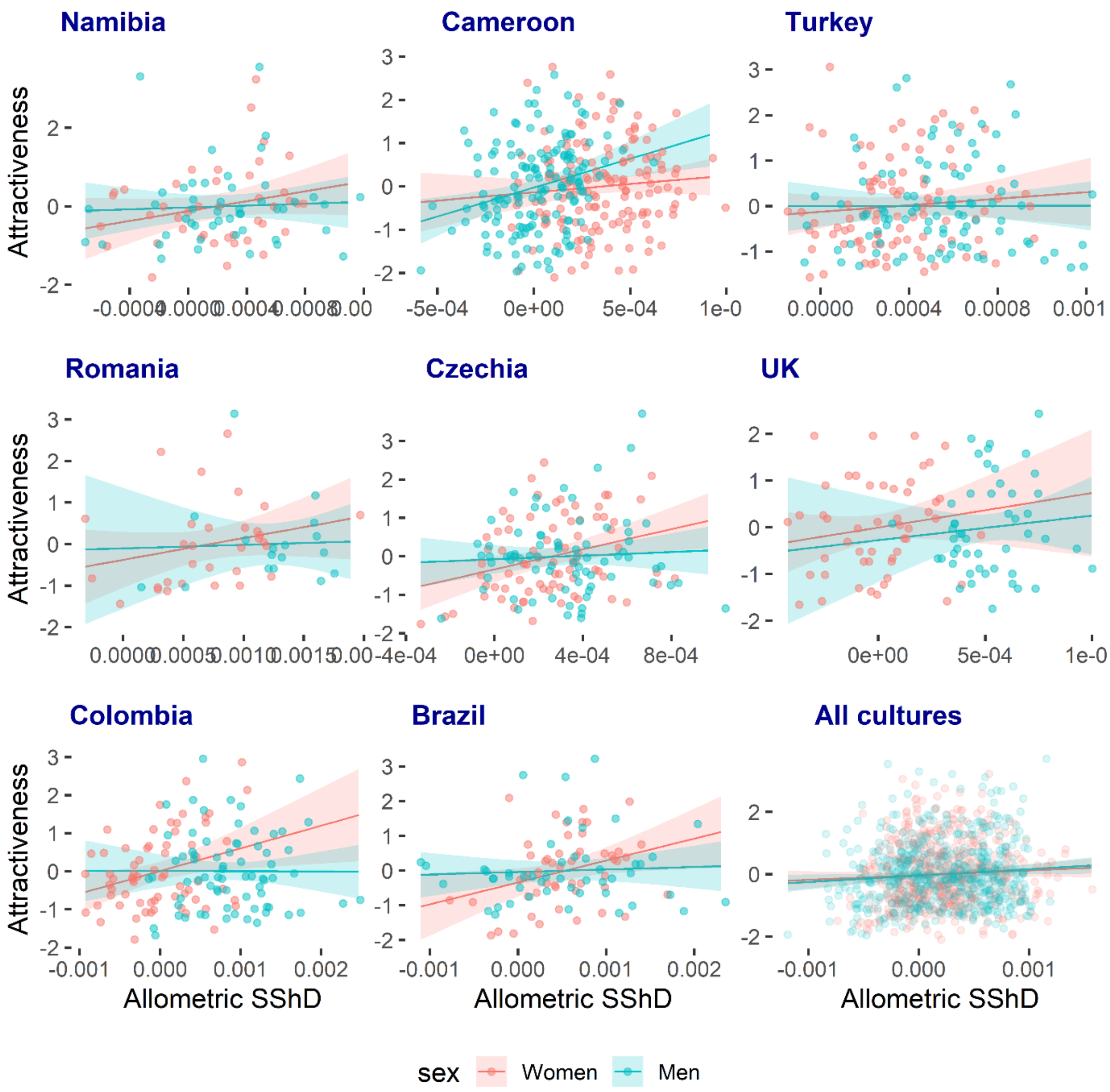

Figure S9. The relationship between allometric SShD and rated facial attractiveness in each population

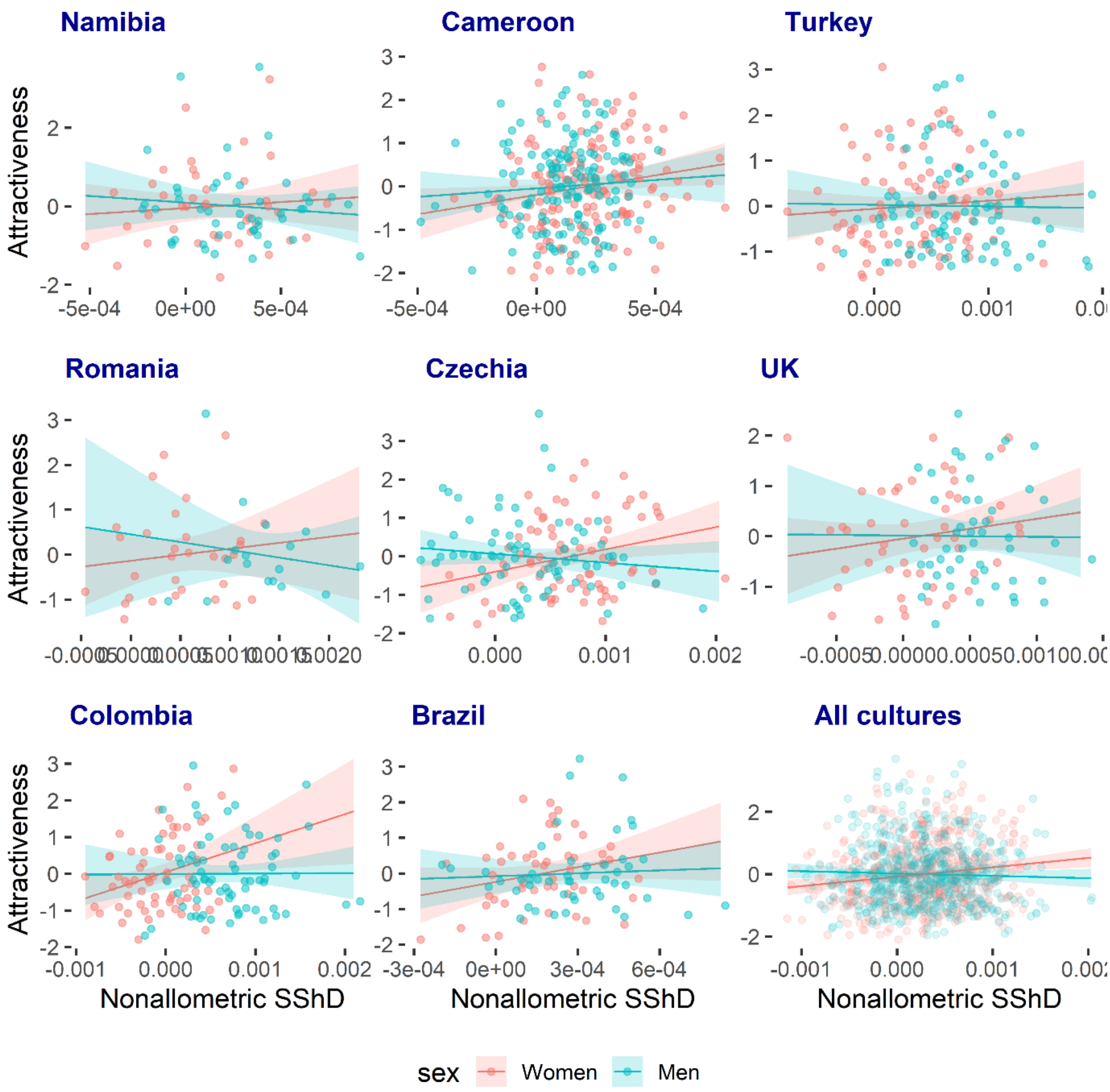

Figure S10. The relationship between nonallometric SShD and rated facial attractiveness in each population

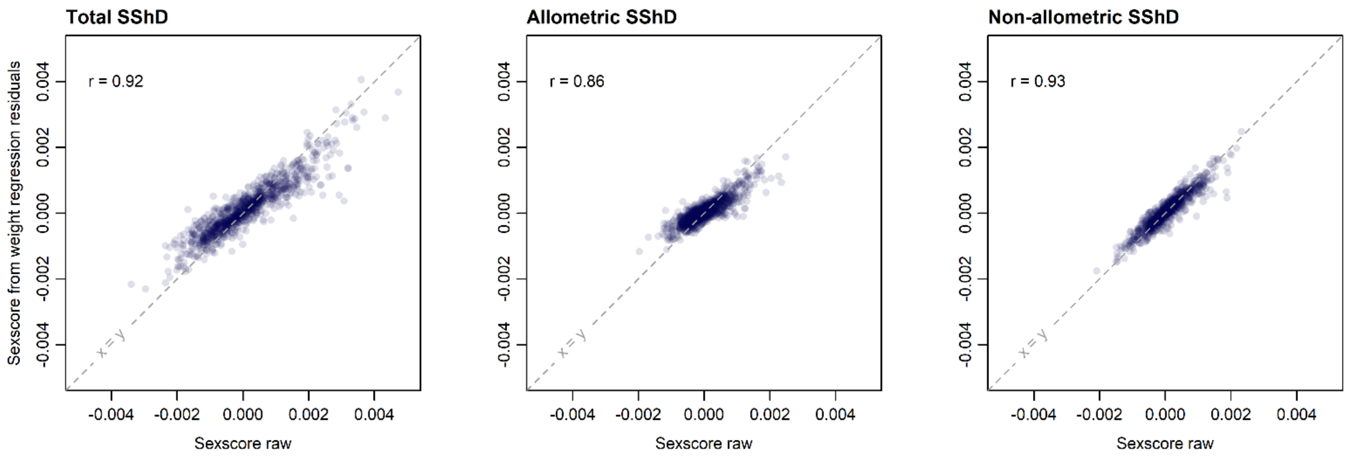

Figure S11. Comparison of the raw sexscores used in the manuscript with the sexcores calculated from residual facial shapes after conditioning on body weight. These sexscores (that agree well with the original measures used because the UK data do not contain information on body weight) are used in an alternative analysis available at https://osf.io/ydkat/

Table S6: Trajectory analysis, path distances (1000 permutations)

Observed path distance		Pairwise absolute differences in path distances				
		contrast	d	UCL (95%)	Z	p
BRAZ	0.039	BRAZ:CMR	0.012	0.008	3.755	0.003
CMR	0.027	BRAZ:COL	0.000	0.009	-1.288	0.990
COL	0.039	BRAZ:CZ	0.001	0.008	-1.129	0.902
CZ	0.039	BRAZ:NAM	0.010	0.009	1.987	0.042
NAM	0.029	BRAZ:RO	0.022	0.012	4.658	0.001
RO	0.061	BRAZ:TR	0.006	0.008	0.939	0.163
TR	0.045	BRAZ:UK	0.004	0.009	0.166	0.375
UK	0.035	CMR:COL	0.012	0.007	4.384	0.001
		CMR:CZ	0.013	0.007	4.866	0.002
		CMR:NAM	0.002	0.008	-0.345	0.542
		CMR:RO	0.035	0.012	7.857	0.001
		CMR:TR	0.018	0.006	7.468	0.001
		CMR:UK	0.008	0.008	1.909	0.048
		COL:CZ	0.000	0.007	-1.109	0.902
		COL:NAM	0.010	0.009	2.163	0.040
		COL:RO	0.022	0.012	4.500	0.002
		COL:TR	0.006	0.008	1.144	0.134
		COL:UK	0.004	0.009	0.274	0.325
		CZ:NAM	0.010	0.009	2.469	0.027
		CZ:RO	0.022	0.012	4.527	0.002
		CZ:TR	0.005	0.007	0.946	0.166
		CZ:UK	0.005	0.009	0.484	0.275
		NAM:RO	0.032	0.013	6.794	0.001
		NAM:TR	0.015	0.008	4.544	0.001
		NAM:UK	0.005	0.009	0.539	0.263
		RO:TR	0.017	0.012	3.198	0.007
		RO:UK	0.027	0.012	5.561	0.001
		TR:UK	0.010	0.008	2.625	0.016

UCL = Upper Compatibility Limit for H0 (no difference)

Table S7. Pairwise correlations between trajectories and respective angles (1000 permutations)

	r	angle	UCL (95%)	Z	p
BRAZ:CMR	0.662	48.544	29.692	5.246	0.001
BRAZ:COL	0.716	44.296	33.486	3.486	0.004
BRAZ:CZ	0.900	25.801	33.392	0.548	0.249
BRAZ:NAM	0.797	37.129	37.403	1.757	0.053
BRAZ:RO	0.636	50.469	44.492	2.590	0.021
BRAZ:TR	0.911	24.402	32.352	0.422	0.281
BRAZ:UK	0.881	28.250	36.091	0.529	0.266
CMR:COL	0.390	67.077	26.789	10.062	0.001
CMR:CZ	0.664	48.365	25.890	6.690	0.001
CMR:NAM	0.774	39.306	30.953	3.255	0.007
CMR:RO	0.677	47.365	39.536	3.035	0.005
CMR:TR	0.735	42.699	24.278	6.240	0.001
CMR:UK	0.816	35.298	30.112	2.897	0.013
COL:CZ	0.762	40.337	30.005	3.749	0.006
COL:NAM	0.653	49.218	34.675	4.153	0.001
COL:RO	0.051	87.049	43.034	7.352	0.001
COL:TR	0.558	56.066	29.651	6.739	0.001
COL:UK	0.453	63.051	33.979	6.611	0.001
CZ:NAM	0.726	43.442	34.088	3.310	0.006
CZ:RO	0.533	57.823	41.747	3.999	0.001
CZ:TR	0.779	38.847	29.386	3.926	0.001
CZ:UK	0.830	33.928	33.636	1.947	0.044
NAM:RO	0.460	62.595	45.848	3.993	0.003
NAM:TR	0.865	30.106	32.666	1.309	0.093
NAM:UK	0.772	39.442	36.841	2.239	0.033
RO:TR	0.735	42.664	42.258	2.034	0.046
RO:UK	0.807	36.208	44.703	0.767	0.207
TR:UK	0.872	29.291	32.875	1.311	0.101

UCL = Upper Compatibility Limit for H0 (the same direction)
